# Structure of the human transcobalamin beta domain in four distinct states

**DOI:** 10.1371/journal.pone.0184932

**Published:** 2017-09-14

**Authors:** Joël S. Bloch, Markus Ruetz, Bernhard Kräutler, Kaspar P. Locher

**Affiliations:** 1 Department of Biology, Institute of Molecular Biology and Biophysics, ETH Zurich, Zurich, Switzerland; 2 Institute of Organic Chemistry and Center of Molecular Biosciences, University of Innsbruck, Innrain, Innsbruck, Austria; Russian Academy of Medical Sciences, RUSSIAN FEDERATION

## Abstract

Vitamin B12 (cyanocobalamin, CNCbl) is an essential cofactor-precursor for two biochemical reactions in humans. When ingested, cobalamins (Cbl) are transported via a multistep transport system into the bloodstream, where the soluble protein transcobalamin (TC) binds Cbl and the complex is taken up into the cells via receptor mediated endocytosis. Crystal structures of TC in complex with CNCbl have been solved previously. However, the initial steps of holo-TC assembly have remained elusive. Here, we present four crystal structures of the beta domain of human TC (TC-beta) in different substrate-bound states. These include the apo and CNCbl-bound states, providing insight into the early steps of holo-TC assembly. We found that *in vitro* assembly of TC-alpha and TC-beta to a complex was Cbl-dependent. We also determined the structure of TC-beta in complex with cobinamide (Cbi), an alternative substrate, shedding light on the specificity of TC. We finally determined the structure of TC-beta in complex with an inhibitory antivitamin B12 (anti-B12). We used this structure to model the binding of anti-B12 into full-length holo-TC and could rule out that the inhibitory function of anti-B12 was based on an inability to form a functional complex with TC.

## Introduction

**Fig 1:**
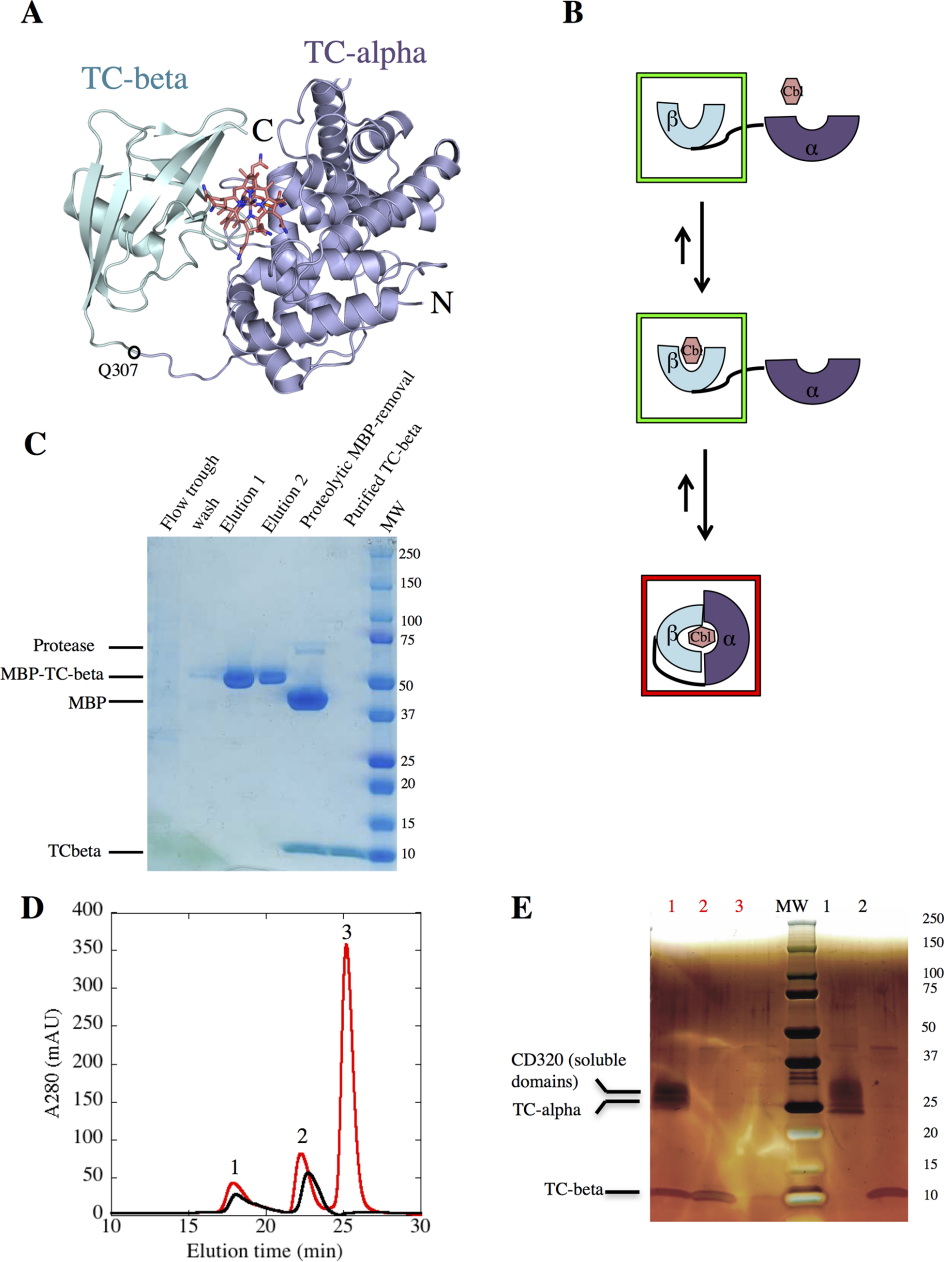
Cbl induced assembly of individually expressed TC domains. (A) PDB entry 4ZRP, holo-TC with the alpha domain (blue) and the beta domain (cyan) in cartoon representation and CNCbl in stick representation (carbons salmon) (receptor CD320 not shown). The TC-beta construct used in this study begins at Q307 and contains additional N-terminal residues from the TEV-cleavage site. (B) Hypothetical stages of TC-alpha, TC-beta assembly upon Cbl addition, green boxes indicate new, red boxes previously published crystal structures. (C) Coomassie stained SDS-PAGE analysis of TC-beta purification. (D) SEC (size exclusion chromatography) profile (TSK-G3000 column) of a mixture of TC-alpha:CD320, TC-beta in the presence (red) or absence (black) of CNCbl of a ratio of 1:4:16 (TC-alpha:TC-beta:CNCbl). (E) Silver stained SDS-PAGE of peak fractions from SEC.

Vitamin B12 (cyanocobalamin, CNCbl) is essential for humans, as at least two biochemical pathways are B12 dependent[1–4]: It is a coenzyme precursor for cytoplasmic methionine synthase and mitochondrial methylmalonyl-CoA mutase. Because only certain prokaryotes are able to synthesize cobalamins (Cbls), humans are dependent on dietary intake of vitamin B12[5]. When ingested, the water-soluble vitamin is transported via a multistep transport system through the ileum into the bloodstream[6]. There, 10–30% of total Cbls are bound to transcobalamin (TC). The remaining Cbl and Cbl analogs are bound to haptocorrin (HC)[7]. Despite having a higher affinity for Cbls[8], HC has a lower substrate specificity than TC and is able to bind various Cbl analogs with similar affinities[9]. Cellular uptake of Cbls occurs via receptor-mediated endocytosis of holo-TC by means of the receptor CD320[10]. The structure of the extracellular domain of CD320 in complex with holo-TC was recently determined[11]. Cancer cells, being in elevated need of Cbls, overexpress the CD320 protein[12]. This makes TC:CD320-mediated uptake of Cbls an interesting target for oncological research. TC consist of two domains (alpha and beta) that are connected by a flexible linker. Both domains contribute to Cbl binding, resulting in sub-pico-molar affinity (Fig 1A) [8, 13]. It has been hypothesized that the two domains only assemble upon binding of Cbls to either one of the two domains (Fig 1B) [13, 14].

To address the question of how Cbl mediated TC assembly proceeds, we determined crystal structures of apo TC-beta and TC-beta in complex with Cbl, putatively representing the first steps of TC assembly. Moreover, we determined the structure of TC-beta in complex with the substrate analog Cbi.

Antivitamins B12 are modified Cbls that are structurally similar to CNCbl, with the β-axial ligand being replaced by inert ligands such as difluorophenylethinyl (S1 Fig) [15–17]. When injected into mice, the anti-B12 compound 4-Ethylphenyl-Cobalamin (EtPhCbl) induced a pronounced Cbl-deficiency[18]. The use of anti-B12 molecules therefore provides a more efficient approach to study the effects of Cbl deficiency than feeding laboratory animals with Cbl-free nutrition, because the latter depends on the slow depletion of the Cbl storage in the liver[19]. Elevated Cbl levels in the blood of mice that have been supplied with EtPhCbl suggest an interference with the cellular uptake of Cbls. The molecular basis of this interference is unknown because it was demonstrated that anti-B12 can bind to TC and can be taken up into cells[18]. To test whether anti-B12 affects Cbl uptake via its interaction with TC, we determined a co-crystal structure of TC-beta bound to Co-β-[2-(2,4- difluorophenyl)ethinyl]cobalamin (F2PhEtyCbl) at high resolution.

## Results

### Expression of human TC-beta and Cbl-dependent assembly of holo-TC

The sub-picomolar affinity of TC to Cbl[6, 20] and the presence of Cbls in eukaryotic expression media made it challenging to produce apo TC without denaturing the protein. We expressed the two domains of TC individually in insect cells, which lowered the affinity for Cbls while retaining the structural integrity of the domains, as no denaturing was required to generate the Cbl-free domains. TC-beta was expressed as a fusion protein with maltose binding protein (MBP). Extensive washing of the immobilized fusion protein was required to completely remove all bound Cbl. After proteolytic cleavage of the MBP and further affinity purification we obtained pure, substrate free TC-beta (Fig 1C). TC-alpha was co-expressed with the soluble/external domains of CD320, as this increased the stability and the yield of the pure protein. TC-alpha:CD320 did not contain bound Cbls after purification, as evidenced by the absence of a peak at 361 nm in the vis-spectrum.

In full-length TC, the alpha- and the beta domains are connected by a flexible linker. In the structure of CNCbl-bound TC, the alpha- and beta domains share a very small binding interface[13]. We therefore wanted to test the hypothesis that the two domains would only assemble upon Cbl binding (Fig 1B). A similar dependence has been observed previously with the alpha- and beta domains of the structurally similar protein intrinsic factor[21].

We incubated TC-beta with TC-alpha:CD320 in the presence or absence CNCbl. Subsequent size exclusion chromatography and SDS-PAGE analysis of the peak fractions revealed that TC-beta and TC-alpha:CD320 only co-eluted when CNCbl was present(Fig 1D and 1E). In contrast, the domains remained separated when no CNCbl was added.

### Crystal structures of TC-beta in apo- and CNCbl-bound form

**Fig 2:**
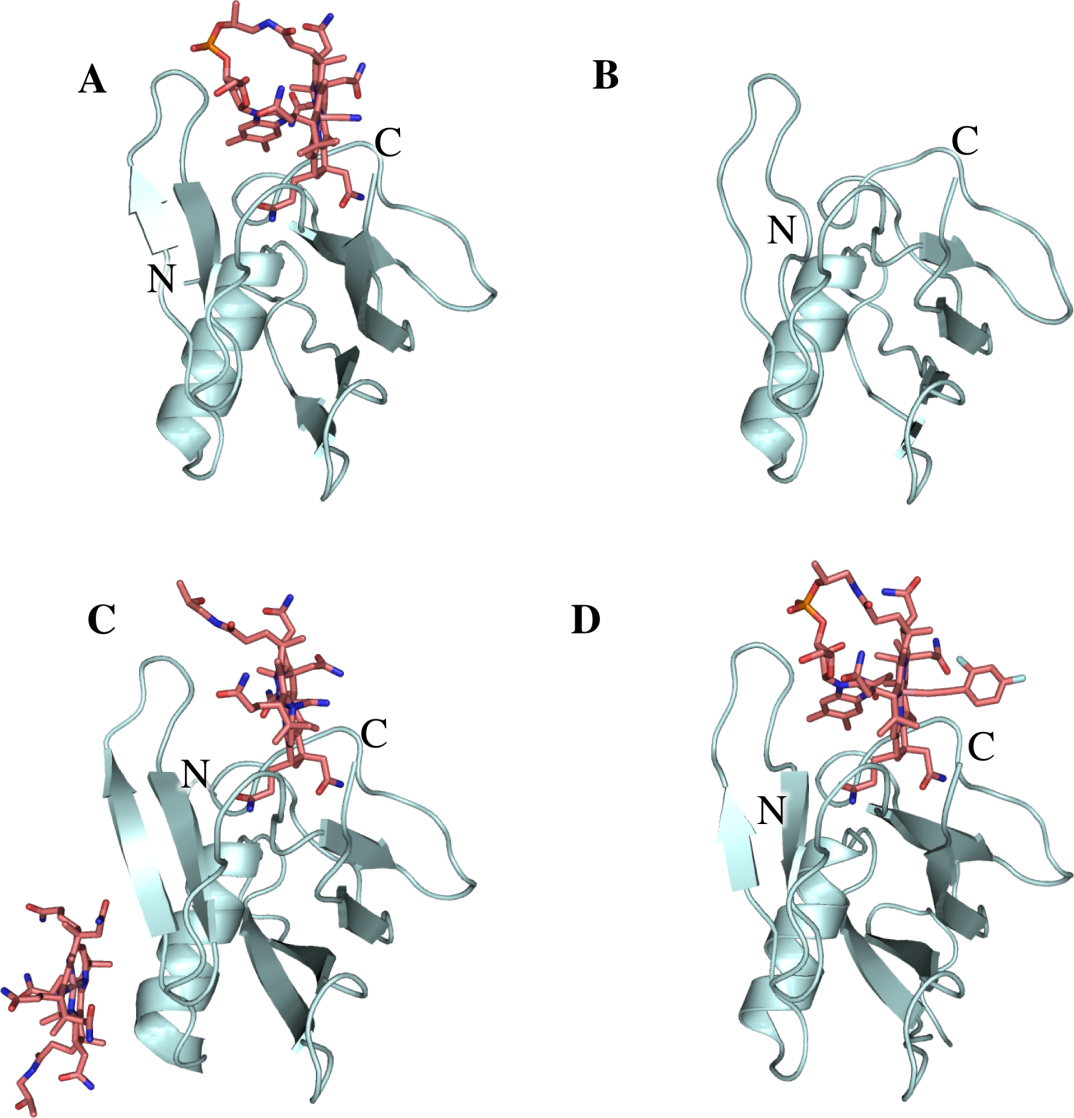
Crystal structures of TC-beta bound to different substrates and in apo form. Crystal structures of TC-beta in four different conformations such as CNCbl-bound TC-beta (A), apo TC-beta (B), Cbi-bound TC-beta (C) and anti-B12-bound TC-beta (D). All 3 ligands bind at the same site of TC-beta in roughly in the same orientation. In the structure of Cbi-bound TC-beta, an additional Cbi is bound to the surface of TC-beta.

**Fig 3:**
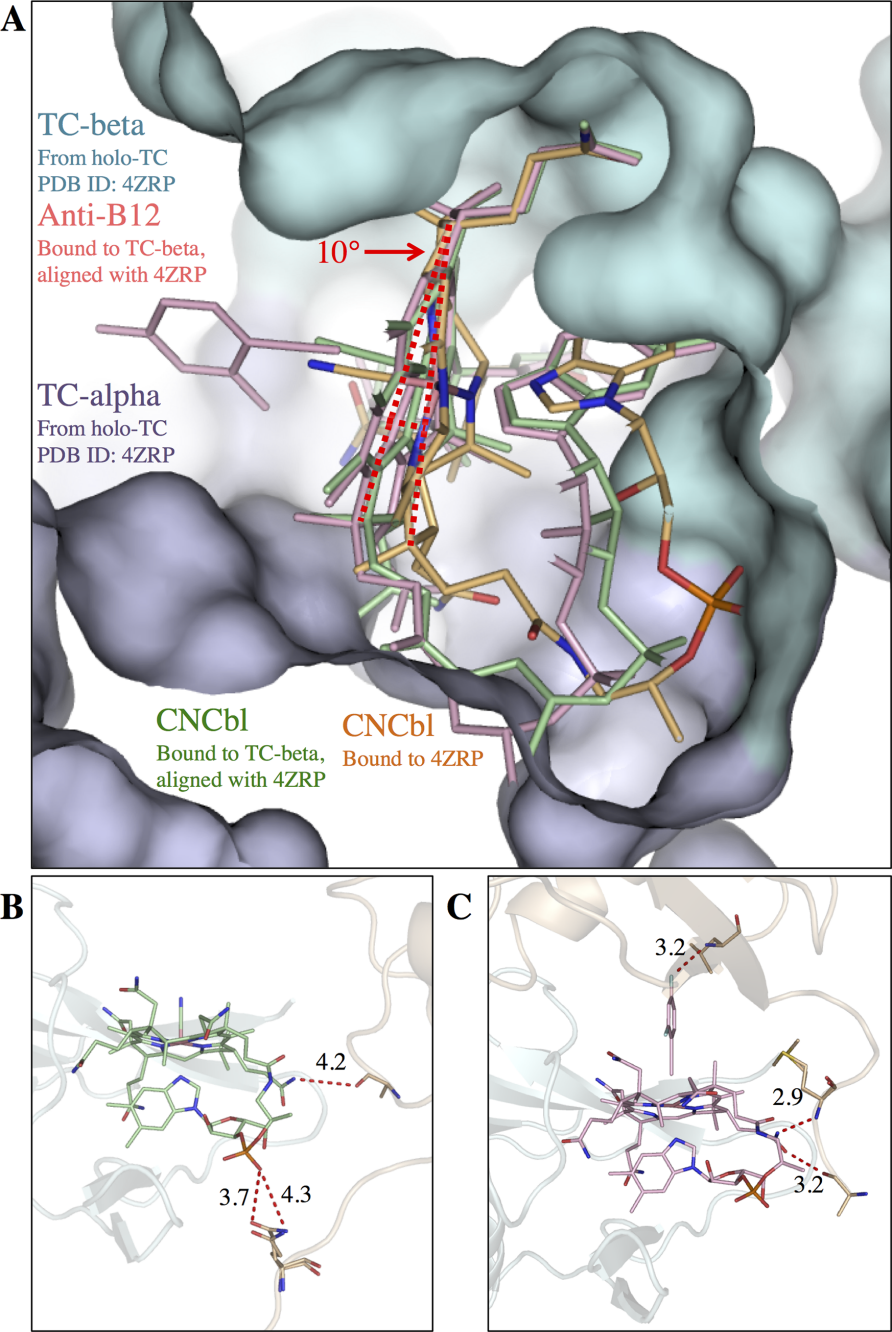
Comparison of ligand positioning in CNCbl-bound TC-beta and full-length TC. (A) Superimposed structures of Cbl-bound TC:CD320 (PDB entry 4ZRP, alpha-domain blue, beta-domain cyan, CNCbl orange, CD320 not shown), CNCbl-bound TC-beta (green, only Cbl shown) and anti-B12-bound TC-beta (pink, only anti-B12 shown). In the absence of TC-alpha, CNCbl and anti-B12 are rotated both by about 10° (indicated by dotted red lines). (B, C) Comparison of the structures of CNCbl-bound TC-beta (B) (cyan, green) and anti-B12-bound TC-beta (C) (cyan, pink) with highlighted polar crystal contacts (dotted red lines) for CNCbl and anti-B12 (symmetry mate in orange).

We first determined the crystal structure CNCbl-bound TC-beta at 1.43 Å resolution (Fig 2A, Table 1). It revealed a highly similar structure compared to the beta domain in full-length holo-TC[11] (rmsd = 0.39 Å for 97 C_α_-atoms, Table 2). However, we observed that CNCbl was rotated out of the binding pocket by 9.2° compared to holo-TC (Fig 3A). As a consequence, not all H-bonds observed in CNCbl-bound TC were formed. For example, the H-bond from the phosphate to the backbone of L358 was missing in the CNCbl-bound TC-beta structure.

**Table 1:** Crystallographic data.

Structure	Apo TC-beta	TC-beta:Cbl	TC-beta:anti-B12	TC-beta:Cbi
**Crystals**	1	1	1	1
**Wavelength (Å)**	1.0	1.0	1.0	1.0
**Resolution range**	41.72–1.57 (1.63–1.57)	30.12–1.43 (1.49–1.43)	43.85–1.27 (1.33–1.27)	41.68–1.57 (1.63–1.57)
**Space group**	P 32 2 1	P 32 2 1	P 21 21 2	P 32 2 1
**Unit cell: a, b, c (Å)**	59.98 59.98 70.04	60.24 60.24 69.57	61.72 62.30 33.76	59.86 59.86 70.11
**α, β, γ (°)**	90 90 120	90 90 120	90 90 90	90 90 120
**Total reflections**	203359 (18492)	257023 (22478)	137130 (7405)	203737 (17928)
**Unique reflections**	20772 (2015)	27022 (2563)	33543 (1161)	19877 (1790)
**Multiplicity**	9.8 (9.2)	9.5 (8.8)	6.3 (6.4)	10.2 (10.0)
**Completeness (%)**	99.76 (98.97)	99.23 (96.61)	96.19 (68.73)	95.53 (87.45)
**Mean I/sigma(I)**	16.80 (0.90)	22.25 (1.03)	24.40 (12.16)	16.79 (0.82)
**R-merge**^a^	0.075 (2.06)	0.046 (1.99)	0.043 (0.11)	0.05742 (2.31)
**CC1/2**	1 (0.33)	1 (0.61)	1 (1.00)	1 (0.52)
CC*	1 (0.70)	1 (0.87)	1 (1.00)	1 (0.83)
**R-work (%)**	15.5 (29.3)	15.6 (32.8)	14.4 (46.9)	17.9 (43.5)
**R-free (%)**	19.9 (32.0)	19.1 (35.6)	17.9 (48.9)	20.8 (45.5)
**Number of non-hydrogen atoms**	1017	1163	1231	1075
**macromolecules**	868	909	892	856
**ligands**	6	99	102	138
**solvent**	143	155	236	81
**Protein residues**	105	106	106	106
**RMS(bonds) (Å)**	0.005	0.008	0.017	0.014
**RMS(angles) (°)**	0.84	1	2.42	1.09
**Ramachandran favored**^b^ **(%)**	97.09	99.04	99	96.15
**Ramachandran allowed**^b^ **(%)**	1.94	0.96	0.96	2.88
**Ramachandran outliers**^b^ **(%)**	0.97	0	0	0.96
**Rotamer outliers**^b^ **(%)**	1.03	0.99	2.04	4.21
**Clashscore**^b^	4.55	10.39	5.64	16.19
**Average B-factor**	36.48	31.65	22.90	46.95
**macromolecules**	32.51	28.05	18.70	43.90
**ligands**	189	40.94	17.60	62.66
**solvent**	54.12	46.87	41.30	52.45

Highest resolution shell in parentheses.

^a^ Rmerge = **∑**hkl |I(hkl)–< I(hkl) > |/ **∑**hkl(hkl), where < I(hkl) > is the mean of the symmetry equivalent reflections of I(hkl).

^b^ As defined in MolProbity.

**Table 2:** RMSD values of TC-beta structures aligned to holo TC.

Protein	Rmsd (Å)
TC-beta:Cbl	0.39
Apo TC-beta	0.47
TC-beta:anti-B12	0.34
TC-beta:Cbi	0.48

Alignment of the TC-beta crystal structures to structure of human TC:CD320 (4KRP) for the residue range 313–409.

We next determined the crystal structure of apo TC-beta at 1.57 Å resolution (Fig 2B, Table 1), which represents to our knowledge the first structure of TC resembling a putative partial apo state of TC in the apo form (i.e. TC-alpha and TC-beta are not associated with each other). The structure was highly similar to the Cbl bound state of TC-beta (rmsd = 0.41 Å for 97 C_α_-atoms, Table 2), with minor changes evident in several side chains compared to CNCbl-bound TC-beta.

### Crystal structure of Cbi-bound TC-beta

Co-crystallization of TC-beta with Cbi yielded crystals diffracting to 1.57 Å resolution (Fig 2C, Table 1). The compound used was dicyano-Cbi, which, when dissolved in water, is converted to the stable monocyano-Co(III)cobinamide. To our surprise, the structure revealed two bound Cbi molecules (Cbi1 and Cbi2). Cbi1 was located in the binding pocket, was bound in a similar way as CNCbl in holo-TC. Notably, the corrin ring/plane of Cbi1 was not rotated compared to the structure of CNCbl-bound full-length TC. We observed a large electron density peak (S2 Fig) in the space occupied by the 5,6-dimethylbenzimidazole (DMB) moiety in Cbl-bound TC. This density was attributed to the N-terminus of a symmetry-related TC-beta molecule in the crystal lattice, with the central cobalt atom of Cbi1 coordinating the side chain of His305. A cyanide ligand bound to the central cobalt atom of Cbi1 could also be modeled (opposite side of His305).

The second Cbi molecule (Cbi2) was found attached to the surface of TC-beta. Its central cobalt atom was coordinated to the side chain of the surface-exposed His345. Here, the histidine was coordinated to the opposite side of the corrin ring compared to Cbi1. Apart from an H-bond to the backbone carbonyl of Tyr352, this was the only contact of Cbi2 to TC-beta. However, Cbi2 was also in contact with a TC-beta symmetry mate. The additional interactions probably caused Cbi2 to be ordered and well-resolved. For Cbi2, no electron density for a bound cyanide ligand was observed, suggesting that it had dissociated. To our knowledge this is the first report of a Cbi molecule non-specifically bound to the surface of a protein.

### Crystal structure of anti-B12-bound TC-beta

**Fig 4:**
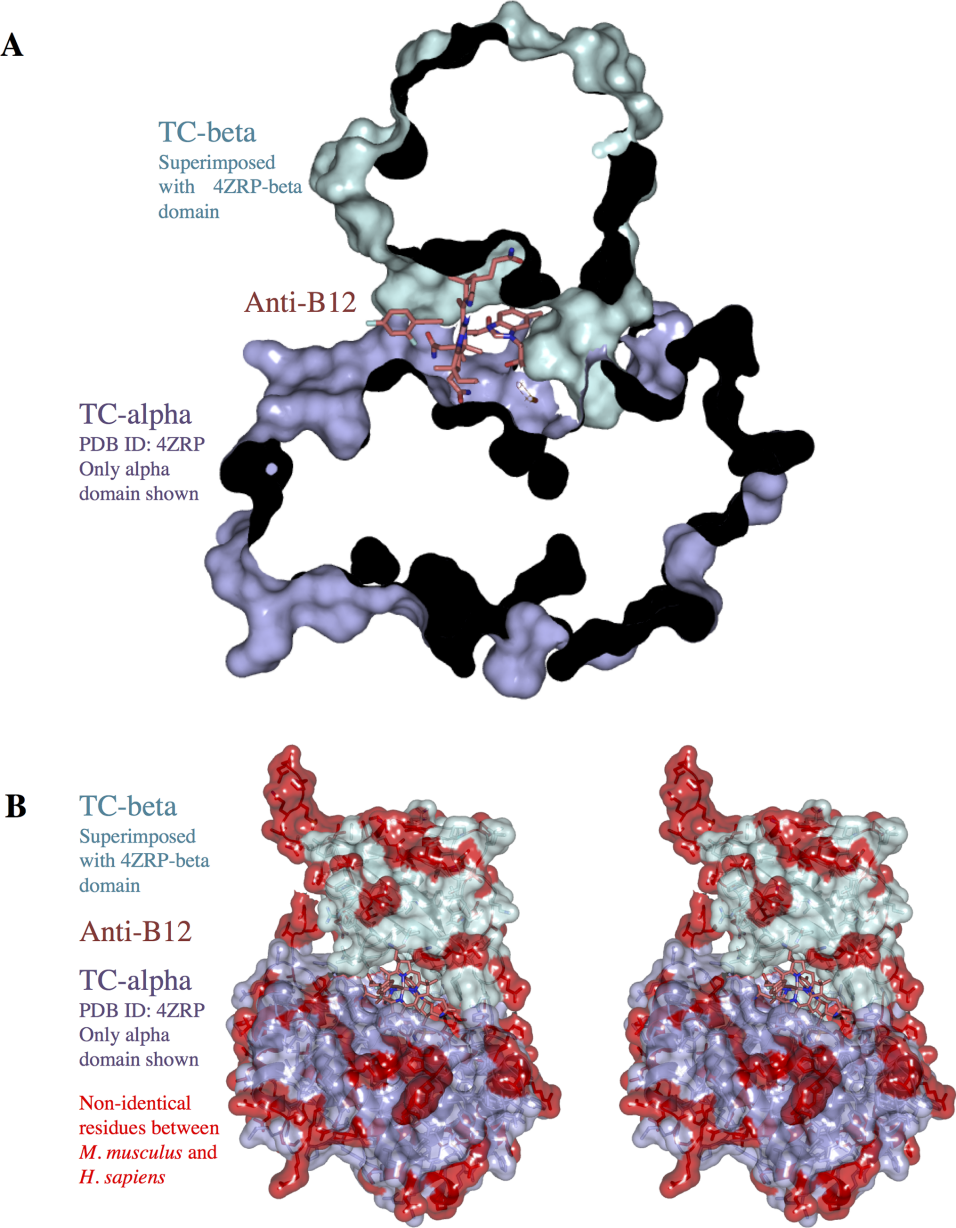
Model of anti-B12 bound to TC. Composite model of the structure of anti-B12-bound TC-beta (salmon, green) superimposed onto CNCbl-bound TC:CD320 (PDB entry 4ZRP, only alpha domain shown, blue). (A) The model shows that anti-B12 bound to TC would not clash with the protein. (B) Stereo representation (wall-eyed), with residues that are not identical between human and mouse TC highlighted in red.

We next determined the crystal structure of TC-beta in complex with the inhibitory B12 analog F2PhEtyCbl (S1 Fig) [17] at 1.27 Å resolution (Fig 2D, Table 1). As apparent in the statistics (high I/σ but low completeness) the crystal would have likely diffracted to higher resolution. However, the data collection was limited by the minimum detector distance of the beamline. The structure and binding mode of anti-B12-bound TC-beta were similar to CNCbl-bound TC-beta (i.e. the anti-B12 was rotated by 11.4° in the same way as CNCbl in TC-beta) (Fig 3A). The F2Ph-group of anti-B12 was clearly visible in the electron density. It was probably well-resolved due to interactions with a symmetry mate in the crystal (S3 Fig). The F2Ph-group was rotated by 90° compared to the small molecule crystal structure of F2PhEty-Cbl [17]. Superposition of the anti-B12-bound TC-beta structure with the crystal structure of CNCbl bound TC:CD320[11] suggested that anti-B12 could bind to TC in the same way as CNCbl and would not generate steric clashes with the F2Ph-β-coaxial ligand (Fig 4).

## Discussion

When we purified full-length TC expressed in insect cells, the protein remained Cbl-bound even after extensive washing on affinity columns (result not shown). For structural studies of the apo state, denaturing poses a potential threat of losing structural integrity. In a previously reported crystal structure of human TC, the disulfide-bridge between Cys65 and Cys78 in the alpha domain was missing[13]. It is conceivable that the denaturing protocol in this study may have resulted in disulfide breakage. To avoid such complications, TC-alpha:CD320 and TC-beta were here expressed individually in insect cells.

Our experiments demonstrate that holo-TC could only be assembled from separated alpha- and beta domains upon addition of a Cbl. This is in line with a previously proposed mechanism of holo-TC assembly[13, 14] and with an earlier finding that the structurally related protein intrinsic factor (IF) only assembles upon CNCbl addition[21].

The structures of apo TC-beta and CNCbl-bound TC-beta provide insight into distinct states of holo-TC assembly. They demonstrate the structural integrity of apo TC-beta and Cbl-bound TC-beta and therefore further consolidate the hypothesized mechanism of Cbl dependent holo-TC assembly. Virtually no structural changes occur in TC-beta upon Cbl binding, arguing against an induced fit mechanism with regards to this domain of TC.

It was shown previously that Cbi can bind to TC and can be transported into cells via CD320-dependent internalization[22]. HC is structurally related to TC and binds Cbi with a much higher affinity: The dissociation rate constants were reported as k_-Cbi_ > 5*10^−2^ s^-1^ for TC and k_-Cbi_ < 1*10^−5^ s^-1^ for HC[8]. In HC, the missing DMB moiety is replaced by Arg-357[23]. Our structure demonstrates that monocyano-Cbi binds to TC-beta in the same way as CNCbl. The lower affinity of TC for Cbi[20] is most likely caused by the reduced number of protein-ligand interactions, given that cobinamides do not contain a DMB moiety.

In Cbi bound TC-beta, both Cbi molecules are coordinated to a histidine, leading to an arrangement where the histidine side chain assumes the role and position of the DMB moiety in Cbl. We hypothesize that cobinamides have the potential to nonspecifically attach to many proteins, as long as they contain solvent-exposed histidines.

When we superimposed the structure of anti-B12-bound TC-beta and Cbl-bound full-length TC[11], it became evident that the anti-B12 would not clash with TC-alpha (Fig 4). There is no structure of CNCbl or anti-B12 bound mouse TC, however the Cbl binding site of human and mouse TC are highly conserved (Fig 4B). Since the receptor-binding site is remote from the substrate-binding site, we believe that the anti-B12 cannot interfere with CD320-mediated uptake of TC. However, anti-B12 will competitively inhibit Cbl binding to TC, which should lead to decreased cellular Cbl uptake. Anti-B12 molecules have been synthesized with an “upper” axial substituent, that is inert to the Cbl-reducing enzyme methylmalonic aciduria and homocystinuria type C protein (CblC) [17, 24, 25]. It remains unclear whether the chemical inertness of anti-B12 molecules has an effect on cellular Cbl uptake, such as for example the down-regulation of CD320 expression. The 90° rotation of the F2Ph-group in our anti-B12-TC-beat structure compared to the conformation observed in the pure compound suggests that this ligand freely rotates and that non-covalent contacts, as provided by a symmetry mate of TC-beta in our structure, determine its conformation (S2 Fig).

In the structures of CNCbl- and anti-B12-bound TC-beta, the corrin planes of the ligands are rotated relative to CNCbl in full-length holo-TC by ~ 10°. We hypothesize that during holo-TC assembly, TC-alpha pushes the substrate deeper into the binding pocket. A crystal contact bias of these structures is unlikely because although both ligands are involved in lattice contacts, the two protein complexes have crystallized in different space groups and feature different lattice contacts (Fig 3B and 3C, Table 1).

Finally, our crystal structures provide a visualization of the putative early steps of holo-TC assembly and into the substrate affinity of TC. With the structure of anti-B12-bound TC-beta we were able to shed light on the mechanism of anti-B12-mediated inhibition of Cbl-uptake. Lastly, we demonstrated that the production of apo TC-beta is a robust method to probe the interaction of TC with alternative substrates, as it is easy to produce and to co-crystallize with various substrates. This could be of great use for the development of drugs targeting TC:CD320-mediated uptake of Cbl. For example, Technetium-99m labeled Cbl analogs were synthesized in the past to target TC mediated Cbl uptake in mice and binding of the compounds to TC as well as tumor labeling was demonstrated successfully[26].

## Experimental procedures

### Expression and purification of TC-beta

All proteins where recombinantly expressed in SF21 cells as described previously for TC:CD320[11]. TC-beta was fused to an N-terminal 9-histidine tag, followed by maltose-binding-protein and a TEV-cleavage site (N-9His-MBP-TEV_cleavage_site-ValAspHisMet-TC-beta(Gln307-W409)). The proteins were purified as described for holo-TC:CD320 previously[11]. For TC-beta an additional washing step during the Ni-NTA immobilization was introduced to remove all bound Cbl: After washing the immobilized 9His-MBP-TC-beta with 10 column volumes of washing buffer (300 mM NaCl, 40 mM imidazole pH 8.0, 20 mM hepes pH 7.0, 0.5 mM CaCl_2_) the protein was washed with “desalting buffer” (500 cv of 150 mM NaCl, 20 mM Tris-HCl pH 7.5, 0.5 mM CaCl_2)_.

### Assembly of TC-alpha:CD320 and TC-beta

10 μM TC-alpha:CD320 in desalting buffer was mixed with 40 μM apo TC-beta with or without 160 μM CNCbl. All components were kept in desalting buffer. The mixtures were incubated overnight at 4°C. Size exclusion chromatography of the mixtures was performed on a TSK-G3000 column using a 1260 Infinity HPLC System from *Agilent Technologies*. Peak fractions were subsequently analyzed by SDS-PAGE and Silver Stain Plus staining from *Biorad*.

### Crystallization, data collection and processing

TC-beta was concentrated in desalting buffer and subsequently crystallized using vapor diffusion in sitting drops. Crystals were cryo protected in 25% glycerol. Diffraction data were collected at the Swiss Light Source (SLS) at the beamlines X06SA (apo TC-beta, Cbi-bound TC-beta, detector EIGER 16M) and X10SA (CNCbl bound TC-beta and anti-B12 bound TC-beta, detector PILATUS 6M) at 1.0 Å. Data were processed using XDS[27]. Model building was done in Coot[28] and refinements were performed in iterative rounds using PHENIX[29]. Initially, XYZ coordinates, Occupancies and Individual B-factors were refined. After every round of refinement the structures were manually improved in Coot [28]. Waters were added initially automatically and afterwards manually. One glycerol molecule was added manually to the structures TC-beta:B12 and apo TC-beta. In the final stages of the refinement, anisotropic B-factor refinement was performed with all structures. Moreover, the X-ray/stereochemistry weight, as well as the X-ray/ADP weights were optimized.

Apo TC-beta was concentrated to 5 mg/ml and was mixed 2:1 with 0.2 M sodium chloride, 20% w/v polyethylene glycol 3,350. The structure was solved by molecular replacement (MR) with PHENIX Phaser-MR[29] using a truncated version of TC (PDB ID: 4ZRP) as search model. Hereby, the residues 1 to 306 as well as the protein CD320 were removed from the model.

For the crystallization of TC-beta:CNCbl, TC-beta was mixed with a 1.5 molar excess of CNCbl to a final concentration of 5 mg/ml and was subsequently mixed 1:1 with 0.15 M Mg formate dihydrate, 22.5% w/v polyethylene glycol 1,000. The structure was solved by MR using the same truncated version of TC that was used for the structure solving of apo TC-beta as search model. The ligand, as well as the geometry-restraints replaced with the files from PDB ID 1CCW [30].

For the crystallization of TC-beta:Cbi_2_, TC-beta was mixed with a 1.5 molar excess of dicyano-Cbi to a final concentration of 5 mg/ml and was subsequently mixed 1:1 with 0.1 M sodium malonate pH 7.0, 12% w/v polyethylene glycol 3,350. The structure was solved by MR using the same truncated version of TC that was used for the structure solving of apo TC-beta as search model. The ligand structure and geometry-restraints files were downloaded from PDB ID 5M29 [31]. In the structure of TC-beta:AntiB12 one calcium molecules was placed, coordinated by one His305 and its backbone amide, which is part of the flexible N-terminus, as well as by two water molecules and the surface exposed His345 of a symmetry mate. A strong electron density peak that is still visible at >18 σ indicated need to place an ion at this position. All other ions that were present in the buffer did either clash with the coordinating atoms due to a too large atomic radius or contained too many electrons which was observed by a large negative electron density peak after refinement.

For the crystallization of TC-beta:anti-B12, TC-beta was mixed with a 1.5 molar excess of F2PhEtyCbl to a final concentration of 5 mg/ml and was subsequently mixed 1:1 with 0.3 M potassium formate, 17% w/v polyethylene glycol 3,350. The structure was solved by MR using the crystal structure apo TC-beta (PDB ID: 5NO0) as search model. The ligand structure and geometry-restraints files were downloaded from PDB ID 5UOS [17].

All superposition calculations were performed using CCP4 Superpose[32] with the option “superpose using secondary structure matching”. For the superposition and rmsd calculations we always used residues 312 to 409 (human TC and TC-beta have the same numbering).

## Supporting information

S1 Fig Chemical formula of cobalamins (vitamin B12 and antivitamins B12). **“**L” indicates the ligand in the structure.
(TIF)

S2 Fig Cbi binding to histidines in TC-beta. TC-beta in complex with Cbi interacting with symmetry mates in the crystal lattice. The blue mesh represents a composite omit map contoured at 1 σ. (A) Cbi1 (pink) in the TC-beta binding pocket (pink), being coordinated to the side-chain of His305 of a symmetry mate (cyan). (B) Cbi2 (salmon) attached to the surface of TC-beta (cyan), being coordinated to the side-chain of His345. Symmetry mates contacting Cbi2 are shown in green and orange.
(TIF)

S3 Fig Crystal contacts of anti-B12 bound to TC-beta. (A) Interaction of TC-beta:anti-B12 (cyan, salmon) with a symmetry-mate (orange) in the crystal. The β-coaxial ligand of anti-B12 is involved in a crystal contact. This interaction probably causes the DFP-group to be ordered in the crystal. (B) Wall-eyed stereo representation of TC-beta:anti-B12. Two TC-beta molecules are shown as sticks, the view is as in A. The blue mesh represents a composite 2Fo-Fc electron density omit map contoured at 2 σ and is shown only around the anti-B12 molecule.
(TIF)

S1 File PDB validation report 5NO0. PDP validation report for the crystal structure of apo TC-beta.
(PDF)

S2 File PDB validation report 5NP4. PDB validation report for the crystal structure of CNCbl-bound TC-beta.
(PDF)

S3 File PDB validation report 5NRP. PDB validation report for the crystal structure of Cbi-bound TC-beta.
(PDF)

S4 File PDB validation report 5NSA. PDB validation report for the crystal structure of anti-B12-bound TC-beta.
(PDF)
