# Supplementary figures and images for: Structure of the human transcobalamin beta domain in four distinct states
Source: PLoS One. 2017 Sep 14;12(9):e0184932. doi: 10.1371/journal.pone.0184932 (PMC5599065; doi:10.1371/journal.pone.0184932)

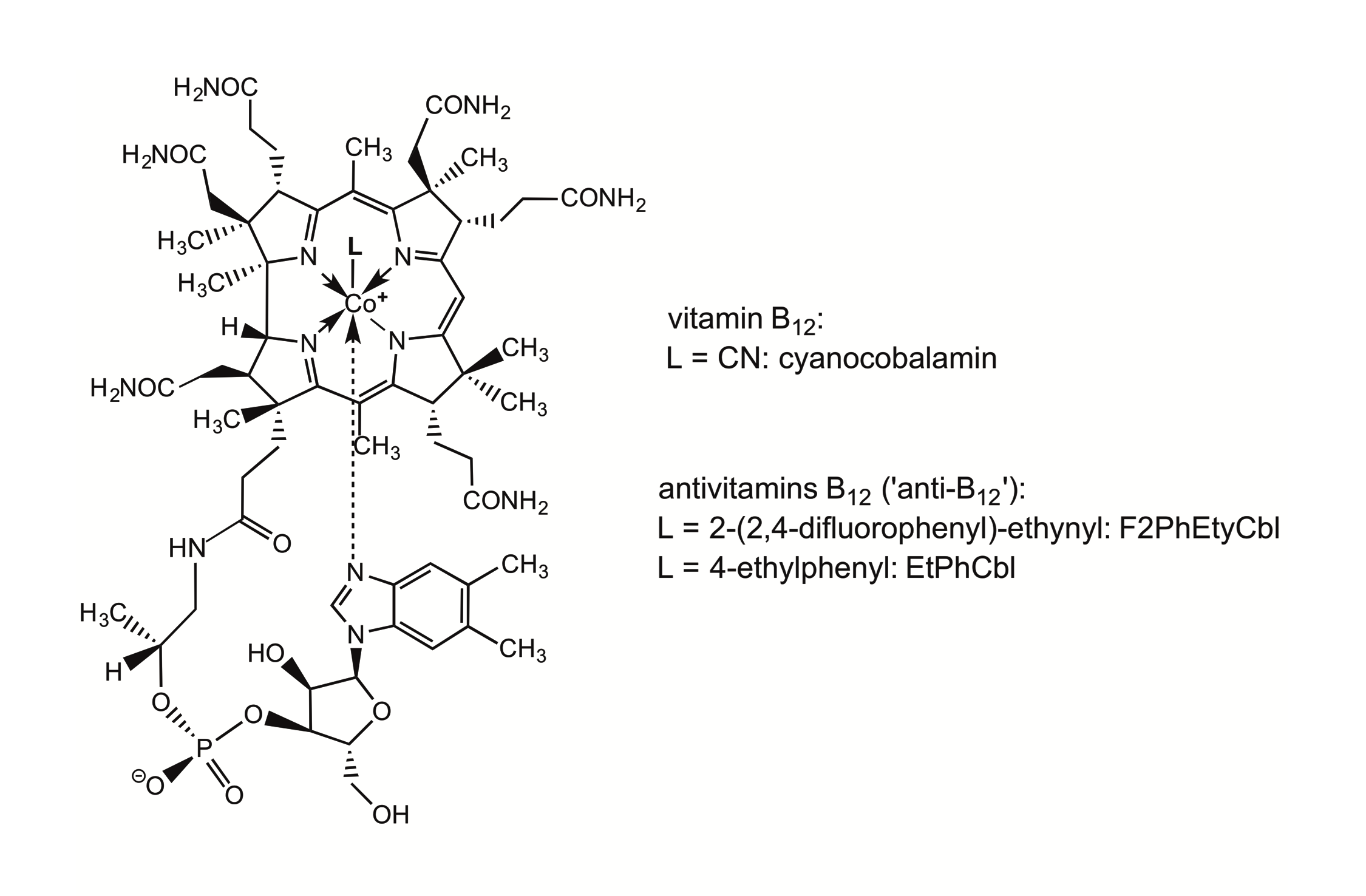

Supplement: S1 Fig — “L” indicates the ligand in the structure. (TIF) [file pone.0184932.s001.tif]

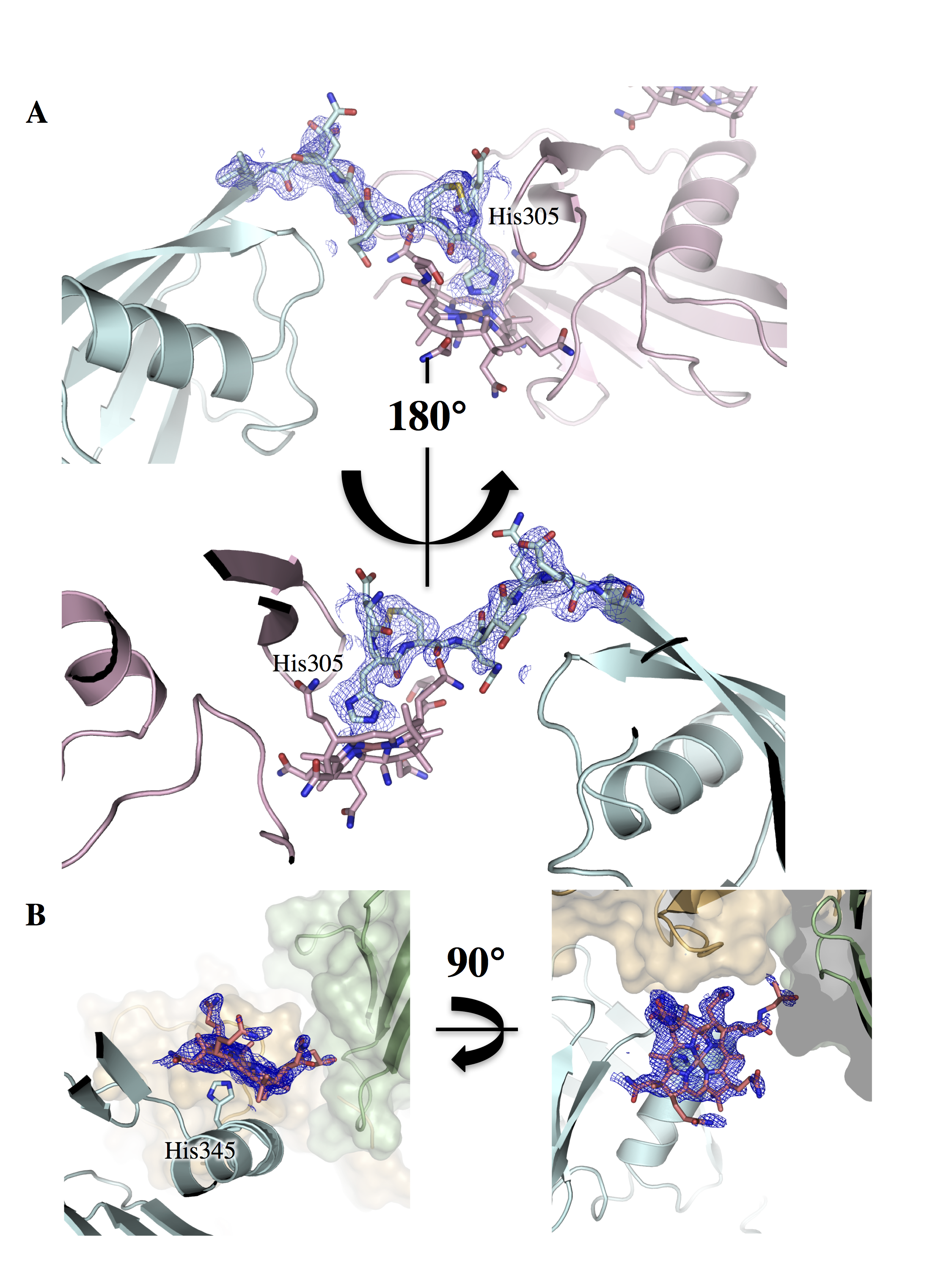

Supplement: S2 Fig — TC-beta in complex with Cbi interacting with symmetry mates in the crystal lattice. The blue mesh represents a composite omit map contoured at 1 σ. (A) Cbi1 (pink) in the TC-beta binding pocket (pink), being coordinated to the side-chain of His305 of a symmetry mate (cyan). (B) Cbi2 (salmon) attached to the surface of TC-beta (cyan), being coordinated to the side-chain of His345. Symmetry mates contacting Cbi2 are shown in green and orange. (TIF) [file pone.0184932.s002.tif]

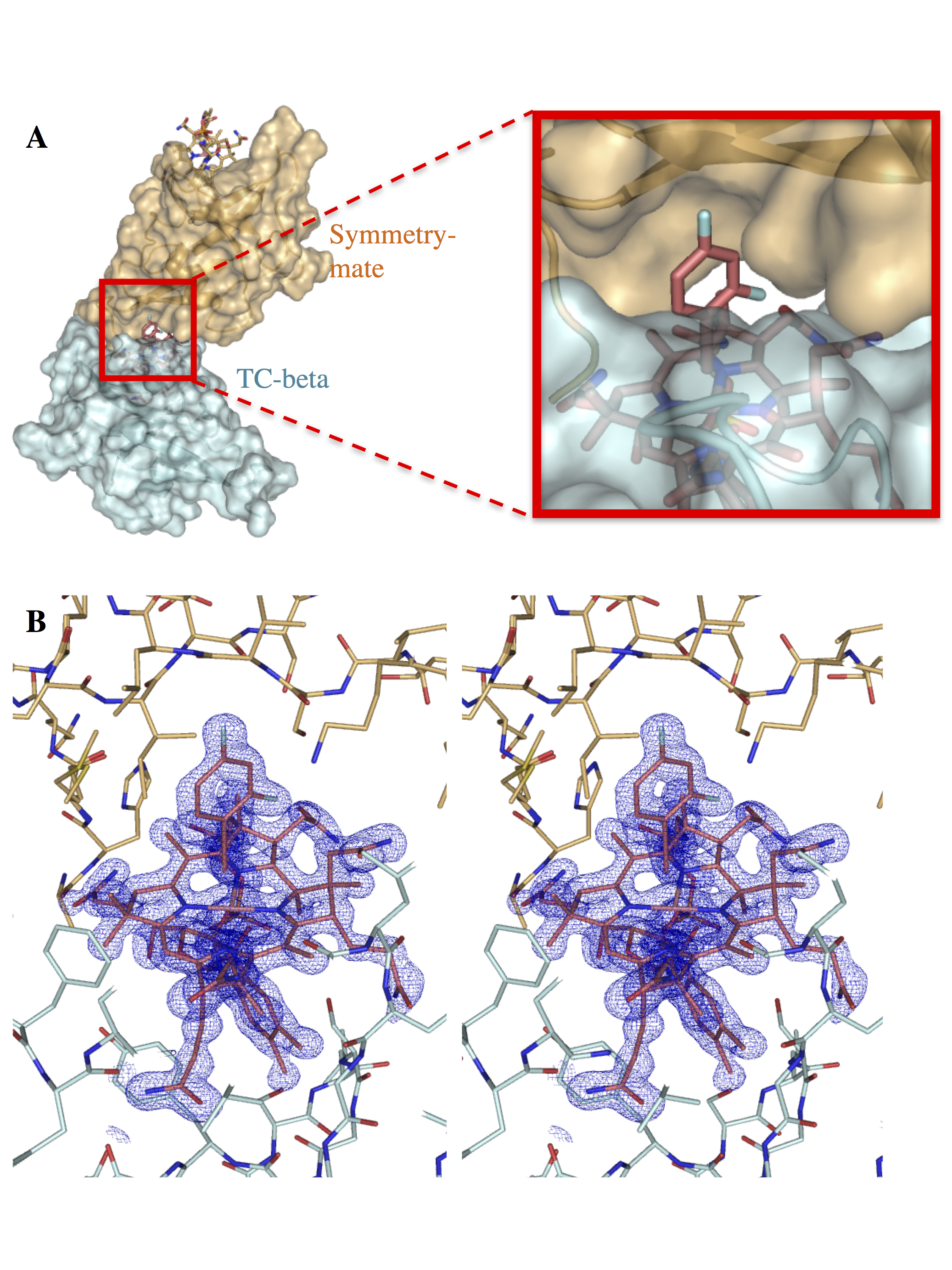

Supplement: S3 Fig — (A) Interaction of TC-beta:anti-B12 (cyan, salmon) with a symmetry-mate (orange) in the crystal. The β-coaxial ligand of anti-B12 is involved in a crystal contact. This interaction probably causes the DFP-group to be ordered in the crystal. (B) Wall-eyed stereo representation of TC-beta:anti-B12. Two TC-beta molecules are shown as sticks, the view is as in A. The blue mesh represents a composite 2Fo-Fc electron density omit map contoured at 2 σ and is shown only around the anti-B12 molecule. (TIF) [file pone.0184932.s003.tif]
