# Supplementary material for: Structure of the human transcobalamin beta domain in four distinct states
Source: PLoS One. 2017 Sep 14;12(9):e0184932. doi: 10.1371/journal.pone.0184932 (PMC5599065; doi:10.1371/journal.pone.0184932)
Supplement: S4 File — PDB validation report for the crystal structure of anti-B12-bound TC-beta. (PDF) [file pone.0184932.s007.pdf]

Full wwPDB X-ray Structure Validation Report (i)

Aug 25, 2017 – 03:38 PM BST

PDB ID	:	5NSA
Title	:	Beta domain of human transcobalamin bound to Co-beta-[2-(2,4-difluorophenyl)ethinyl]cobalamin
Deposited on	:	2017-04-25
Resolution	:	1.27 Å(reported)

This is a Full wwPDB X-ray Structure Validation Report.

This report is produced by the wwPDB biocuration pipeline after annotation of the structure.

We welcome your comments at *validation@mail.wwpdb.org*
A user guide is available at
http://wwpdb.org/validation/2016/XrayValidationReportHelp
with specific help available everywhere you see the (i) symbol.

CONFIDENTIAL VALIDATION REPORT

The following versions of software and data (see references (i)) were used in the production of this report:

MolProbity	:	4.02b-467
Mogul	:	1.7.2 (RC1), CSD as538be (2017)
Xtriage (Phenix)	:	1.9-1692
EDS	:	rb-20029824
Percentile statistics	:	20161228.v01 (using entries in the PDB archive December 28th 2016)
Refmac	:	5.8.0135
CCP4	:	6.5.0
Ideal geometry (proteins)	:	Engh & Huber (2001)
Ideal geometry (DNA, RNA)	:	Parkinson et al. (1996)
Validation Pipeline (wwPDB-VP)	:	rb-20029824

1 Overall quality at a glance ⓘ

The following experimental techniques were used to determine the structure:
X-RAY DIFFRACTION

The reported resolution of this entry is 1.27 Å.

Percentile scores (ranging between 0-100) for global validation metrics of the entry are shown in the following graphic. The table shows the number of entries on which the scores are based.

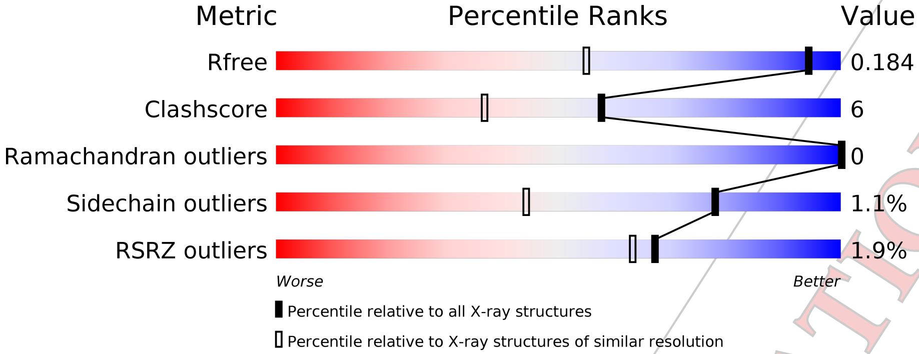

Metric	Whole archive (#Entries)	Similar resolution (#Entries, resolution range(Å))
R_{free}	100719	1367 (1.30-1.26)
Clashscore	112137	1447 (1.30-1.26)
Ramachandran outliers	110173	1392 (1.30-1.26)
Sidechain outliers	110143	1391 (1.30-1.26)
RSRZ outliers	101464	1370 (1.30-1.26)

The table below summarises the geometric issues observed across the polymeric chains and their fit to the electron density. The red, orange, yellow and green segments on the lower bar indicate the fraction of residues that contain outliers for >=3, 2, 1 and 0 types of geometric quality criteria. A grey segment represents the fraction of residues that are not modelled. The numeric value for each fraction is indicated below the corresponding segment, with a dot representing fractions <=5% The upper red bar (where present) indicates the fraction of residues that have poor fit to the electron density. The numeric value is given above the bar.

Mol	Chain	Length	Quality of chain
1	A	108	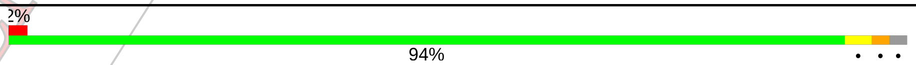

CONFIDENTIAL VALIDATION REPORT

2 Entry composition (i)

There are 5 unique types of molecules in this entry. The entry contains 2200 atoms, of which 969 are hydrogens and 0 are deuteriums.

In the tables below, the ZeroOcc column contains the number of atoms modelled with zero occupancy, the AltConf column contains the number of residues with at least one atom in alternate conformation and the Trace column contains the number of residues modelled with at most 2 atoms.

- Molecule 1 is a protein called Transcobalamin-2.

Mol	Chain	Residues	Atoms						ZeroOcc	AltConf	Trace
			Total	C	H	N	O	S			
1	A	106	1771	565	879	149	174	4	20	14	0

There are 5 discrepancies between the modelled and reference sequences:

Chain	Residue	Modelled	Actual	Comment	Reference
A	302	GLY	-	expression tag	UNP P20062
A	303	VAL	-	expression tag	UNP P20062
A	304	ASP	-	expression tag	UNP P20062
A	305	HIS	-	expression tag	UNP P20062
A	306	MET	-	expression tag	UNP P20062

- Molecule 2 is COBALAMIN (three-letter code: B12) (formula: $C_{62}H_{89}CoN_{13}O_{14}P$).

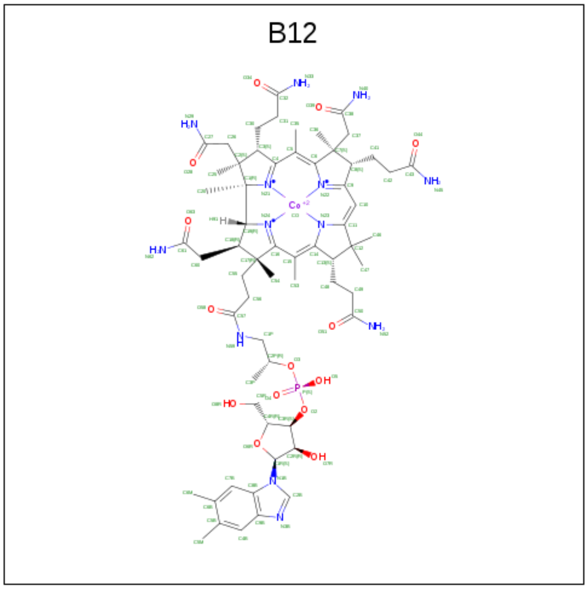

Mol	Chain	Residues	Atoms							ZeroOcc	AltConf
			Total	C	Co	H	N	O	P		
2	A	1	178	62	1	87	13	14	1	0	0

VALIDATION REPORT

- Molecule 3 is 1-ethynyl-2,4-difluorobenzene (three-letter code: 8FS) (formula: $C_8H_4F_2$).

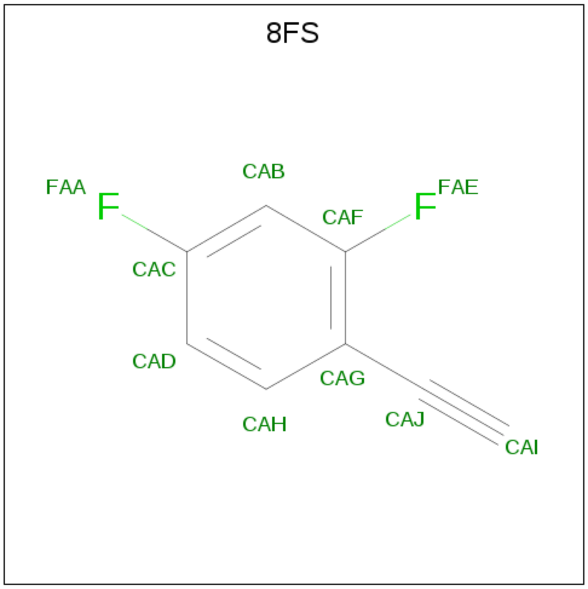

Mol	Chain	Residues	Atoms	ZeroOcc	AltConf
3	A	1	Total C F H<br>13 8 2 3	0	0

- Molecule 4 is CALCIUM ION (three-letter code: CA) (formula: Ca).

Mol	Chain	Residues	Atoms	ZeroOcc	AltConf
4	A	1	Total Ca<br>1 1	0	0

- Molecule 5 is water.

Mol	Chain	Residues	Atoms	ZeroOcc	AltConf
5	A	236	Total O<br>237 237	0	5

CONFIDENTIAL VALIDATION REPORT

3 Residue-property plots (i)

These plots are drawn for all protein, RNA and DNA chains in the entry. The first graphic for a chain summarises the proportions of the various outlier classes displayed in the second graphic. The second graphic shows the sequence view annotated by issues in geometry and electron density. Residues are color-coded according to the number of geometric quality criteria for which they contain at least one outlier: green = 0, yellow = 1, orange = 2 and red = 3 or more. A red dot above a residue indicates a poor fit to the electron density (RSRZ > 2). Stretches of 2 or more consecutive residues without any outlier are shown as a green connector. Residues present in the sample, but not in the model, are shown in grey.

- Molecule 1: Transcobalamin-2

Chain A:

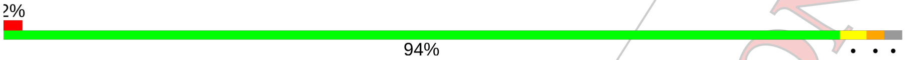

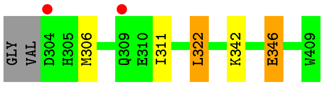

CONFIDENTIAL VALIDATION REPORT

4 Data and refinement statistics (i)

Property	Value	Source
Space group	P 21 21 2	Depositor
Cell constants<br>a, b, c, α, β, γ	61.72Å 62.30Å 33.76Å<br>90.00° 90.00° 90.00°	Depositor
Resolution (Å)	43.85 – 1.27<br>43.85 – 1.27	Depositor<br>EDS
% Data completeness<br>(in resolution range)	94.7 (43.85-1.27)<br>96.2 (43.85-1.27)	Depositor<br>EDS
R_{merge}	(Not available)	Depositor
R_{sym}	(Not available)	Depositor
$< I/\sigma(I) >$ [1]	1.24 (at 1.27Å)	Xtriage
Refinement program	PHENIX (1.12_2829: ???)	Depositor
R, R_{free}	0.144 , 0.179<br>0.147 , 0.184	Depositor<br>DCC
R_{free} test set	1672 reflections (4.99%)	DCC
Wilson B-factor (Å^2)	15.0	Xtriage
Anisotropy	0.151	Xtriage
Bulk solvent k_{sol}(e/Å^3), B_{sol}(Å^2)	0.37 , 49.9	EDS
L-test for twinning[2]	$< \|L\| > = 0.49$, $< L^2 > = 0.32$	Xtriage
Estimated twinning fraction	0.021 for k,h,-l	Xtriage
F_o,F_c correlation	0.97	EDS
Total number of atoms	2200	wwPDB-VP
Average B, all atoms (Å^2)	22.0	wwPDB-VP

Xtriage's analysis on translational NCS is as follows: *The largest off-origin peak in the Patterson function is 10.94% of the height of the origin peak. No significant pseudotranslation is detected.*

[1]Intensities estimated from amplitudes.

[2]Theoretical values of $< |L| >$, $< L^2 >$ for acentric reflections are 0.5, 0.333 respectively for untwinned datasets, and 0.375, 0.2 for perfectly twinned datasets.

CONFIDENTIAL VALIDATION REPORT

5 Model quality (i)

5.1 Standard geometry (i)

Bond lengths and bond angles in the following residue types are not validated in this section: CA, B12, 8FS

The Z score for a bond length (or angle) is the number of standard deviations the observed value is removed from the expected value. A bond length (or angle) with $|Z| > 5$ is considered an outlier worth inspection. RMSZ is the root-mean-square of all Z scores of the bond lengths (or angles).

Mol	Chain	Bond lengths		Bond angles	
		RMSZ	#$\|Z\|$ >5	RMSZ	#$\|Z\|$ >5
1	A	0.56	2/964 (0.2%)	0.77	6/1308 (0.5%)

All (2) bond length outliers are listed below:

Mol	Chain	Res	Type	Atoms	Z	Observed(Å)	Ideal(Å)
1	A	346[A]	GLU	CG-CD	7.85	1.63	1.51
1	A	346[B]	GLU	CG-CD	7.85	1.63	1.51

All (6) bond angle outliers are listed below:

Mol	Chain	Res	Type	Atoms	Z	Observed(^{o})	Ideal(^{o})
1	A	322[A]	LEU	CB-CG-CD2	6.71	122.42	111.00
1	A	322[B]	LEU	CB-CG-CD2	6.71	122.42	111.00
1	A	346[A]	GLU	CG-CD-OE2	-5.44	107.42	118.30
1	A	346[B]	GLU	CG-CD-OE2	-5.44	107.42	118.30
1	A	346[A]	GLU	CG-CD-OE1	5.28	128.86	118.30
1	A	346[B]	GLU	CG-CD-OE1	5.28	128.86	118.30

There are no chirality outliers.

There are no planarity outliers.

5.2 Too-close contacts (i)

In the following table, the Non-H and H(model) columns list the number of non-hydrogen atoms and hydrogen atoms in the chain respectively. The H(added) column lists the number of hydrogen atoms added and optimized by MolProbity. The Clashes column lists the number of clashes within the asymmetric unit, whereas Symm-Clashes lists symmetry related clashes.

CONFIDENTIAL VALIDATION REPORT

Mol	Chain	Non-H	H(model)	H(added)	Clashes	Symm-Clashes
1	A	892	879	840	3	0
2	A	91	87	87	8	0
3	A	10	3	0	0	0
4	A	1	0	0	0	0
5	A	237	0	0	1	1
All	All	1231	969	927	11	1

The all-atom clashscore is defined as the number of clashes found per 1000 atoms (including hydrogen atoms). The all-atom clashscore for this structure is 6.

All (11) close contacts within the same asymmetric unit are listed below, sorted by their clash magnitude.

Atom-1	Atom-2	Interatomic distance (Å)	Clash overlap (Å)
2:A:501:B12:H362	2:A:501:B12:H351	1.47	0.95
2:A:501:B12:H531	2:A:501:B12:H552	1.57	0.85
2:A:501:B12:H531	2:A:501:B12:C55	2.25	0.63
2:A:501:B12:H362	2:A:501:B12:C35	2.29	0.56
2:A:501:B12:H203	2:A:501:B12:H301	1.91	0.51
1:A:306[B]:MET:HE1	1:A:311:ILE:O	2.14	0.47
1:A:306[B]:MET:HE2	5:A:603:HOH:O	2.19	0.43
2:A:501:B12:H351	2:A:501:B12:C36	2.23	0.43
1:A:342:LYS:O	1:A:346[B]:GLU:HG3	2.20	0.42
2:A:501:B12:H262	2:A:501:B12:H601	2.02	0.42
2:A:501:B12:H252	2:A:501:B12:H621	1.86	0.40

All (1) symmetry-related close contacts are listed below. The label for Atom-2 includes the symmetry operator and encoded unit-cell translations to be applied.

Atom-1	Atom-2	Interatomic distance (Å)	Clash overlap (Å)
5:A:828:HOH:O	5:A:828:HOH:O[2_655]	1.95	0.25

5.3 Torsion angles (i)

5.3.1 Protein backbone (i)

In the following table, the Percentiles column shows the percent Ramachandran outliers of the chain as a percentile score with respect to all X-ray entries followed by that with respect to entries of similar resolution.

The Analysed column shows the number of residues for which the backbone conformation was analysed, and the total number of residues.

CONFIDENTIAL VALIDATION REPORT

Mol	Chain	Analysed	Favoured	Allowed	Outliers	Percentiles	
1	A	117/108 (108%)	116 (99%)	1 (1%)	0	100	100

There are no Ramachandran outliers to report.

5.3.2 Protein sidechains (i)

In the following table, the Percentiles column shows the percent sidechain outliers of the chain as a percentile score with respect to all X-ray entries followed by that with respect to entries of similar resolution.

The Analysed column shows the number of residues for which the sidechain conformation was analysed, and the total number of residues.

Mol	Chain	Analysed	Rotameric	Outliers	Percentiles	
1	A	103/93 (111%)	101 (98%)	2 (2%)	62	21

All (2) residues with a non-rotameric sidechain are listed below:

Mol	Chain	Res	Type
1	A	322[A]	LEU
1	A	322[B]	LEU

Some sidechains can be flipped to improve hydrogen bonding and reduce clashes. There are no such sidechains identified.

5.3.3 RNA (i)

There are no RNA molecules in this entry.

5.4 Non-standard residues in protein, DNA, RNA chains (i)

There are no non-standard protein/DNA/RNA residues in this entry.

5.5 Carbohydrates (i)

There are no carbohydrates in this entry.

5.6 Ligand geometry (i)

Of 3 ligands modelled in this entry, 1 is monoatomic - leaving 2 for Mogul analysis.

CONFIDENTIAL VALIDATION REPORT

In the following table, the Counts columns list the number of bonds (or angles) for which Mogul statistics could be retrieved, the number of bonds (or angles) that are observed in the model and the number of bonds (or angles) that are defined in the chemical component dictionary. The Link column lists molecule types, if any, to which the group is linked. The Z score for a bond length (or angle) is the number of standard deviations the observed value is removed from the expected value. A bond length (or angle) with $|Z| > 2$ is considered an outlier worth inspection. RMSZ is the root-mean-square of all Z scores of the bond lengths (or angles).

Mol	Type	Chain	Res	Link	Bond lengths			Bond angles		
					Counts	RMSZ	$\#\|Z\| > 2$	Counts	RMSZ	$\#\|Z\| > 2$
2	B12	A	501	3	73,101,101	1.15	7 (9%)	111,166,166	1.75	10 (9%)
3	8FS	A	502	2	10,10,10	1.87	3 (30%)	13,13,13	1.77	5 (38%)

In the following table, the Chirals column lists the number of chiral outliers, the number of chiral centers analysed, the number of these observed in the model and the number defined in the chemical component dictionary. Similar counts are reported in the Torsion and Rings columns. '-' means no outliers of that kind were identified.

Mol	Type	Chain	Res	Link	Chirals	Torsions	Rings
2	B12	A	501	3	-	0/51/223/223	0/3/11/11
3	8FS	A	502	2	-	0/0/2/2	0/1/1/1

All (10) bond length outliers are listed below:

Mol	Chain	Res	Type	Atoms	Z	Observed(Å)	Ideal(Å)
3	A	502	8FS	CAJ-CAI	-4.20	1.03	1.17
2	A	501	B12	C8B-N1B	-3.85	1.33	1.38
2	A	501	B12	C11-C10	-3.69	1.34	1.41
2	A	501	B12	C2-C3	-2.59	1.54	1.58
2	A	501	B12	C4B-C9B	-2.25	1.38	1.41
2	A	501	B12	C1-C19	-2.18	1.50	1.55
3	A	502	8FS	CAG-CAJ	-2.10	1.40	1.44
3	A	502	8FS	CAD-CAC	2.10	1.41	1.37
2	A	501	B12	C6B-C5B	2.66	1.47	1.41
2	A	501	B12	C8B-C9B	3.72	1.47	1.40

All (15) bond angle outliers are listed below:

Mol	Chain	Res	Type	Atoms	Z	Observed(°)	Ideal(°)
2	A	501	B12	C20-C1-C19	-8.43	101.17	109.34
2	A	501	B12	C18-C60-C61	-5.13	100.65	113.98
2	A	501	B12	C25-C2-C3	-3.24	110.58	115.56
2	A	501	B12	C13-C14-C15	-3.23	120.64	131.85
2	A	501	B12	C9-C10-C11	-2.99	123.17	131.90

Continued on next page...

CONFIDENTIAL VALIDATION REPORT

Continued from previous page...

Mol	Chain	Res	Type	Atoms	Z	Observed(^{o})	Ideal(^{o})
2	A	501	B12	C3-C4-C5	-2.60	122.82	131.85
3	A	502	8FS	CAD-CAC-CAB	-2.34	120.22	123.29
3	A	502	8FS	CAB-CAF-CAG	-2.22	120.10	122.83
3	A	502	8FS	FAE-CAF-CAG	2.02	120.42	117.55
2	A	501	B12	C35-C5-C6	2.08	120.57	117.85
3	A	502	8FS	CAF-CAB-CAC	2.71	119.61	116.65
3	A	502	8FS	CAH-CAG-CAF	3.01	120.37	117.52
2	A	501	B12	C2-C1-C19	4.09	125.17	118.60
2	A	501	B12	C1-C19-C18	5.36	130.84	121.90
2	A	501	B12	C1-C19-N24	9.91	117.55	106.24

There are no chirality outliers.

There are no torsion outliers.

There are no ring outliers.

1 monomer is involved in 8 short contacts:

Mol	Chain	Res	Type	Clashes	Symm-Clashes
2	A	501	B12	8	0

5.7 Other polymers ⓘ

There are no such residues in this entry.

5.8 Polymer linkage issues ⓘ

There are no chain breaks in this entry.

CONFIDENTIAL VALIDATION REPORT

6 Fit of model and data (i)

6.1 Protein, DNA and RNA chains (i)

In the following table, the column labelled '#RSRZ> 2' contains the number (and percentage) of RSRZ outliers, followed by percent RSRZ outliers for the chain as percentile scores relative to all X-ray entries and entries of similar resolution. The OWAB column contains the minimum, median, 95th percentile and maximum values of the occupancy-weighted average B-factor per residue. The column labelled 'Q< 0.9' lists the number of (and percentage) of residues with an average occupancy less than 0.9.

Mol	Chain	Analysed	<RSRZ>	#RSRZ>2			OWAB(Å^2)	Q<0.9
1	A	106/108 (98%)	-0.15	2 (1%)	67	63	12, 17, 29, 35	1 (0%)

All (2) RSRZ outliers are listed below:

Mol	Chain	Res	Type	RSRZ
1	A	309[A]	GLN	2.2
1	A	304	ASP	2.2

6.2 Non-standard residues in protein, DNA, RNA chains (i)

There are no non-standard protein/DNA/RNA residues in this entry.

6.3 Carbohydrates (i)

There are no carbohydrates in this entry.

6.4 Ligands (i)

In the following table, the Atoms column lists the number of modelled atoms in the group and the number defined in the chemical component dictionary. LLDF column lists the quality of electron density of the group with respect to its neighbouring residues in protein, DNA or RNA chains. The B-factors column lists the minimum, median, 95th percentile and maximum values of B factors of atoms in the group. The column labelled 'Q< 0.9' lists the number of atoms with occupancy less than 0.9.

Mol	Type	Chain	Res	Atoms	RSCC	RSR	LLDF	B-factors(Å^2)	Q<0.9
3	8FS	A	502	10/10	0.93	0.08	0.24	19,21,25,27	0
2	B12	A	501	91/91	0.98	0.07	-0.12	11,18,28,39	0
4	CA	A	503	1/1	0.99	0.07	-	15,15,15,15	0

CONFIDENTIAL VALIDATION REPORT

6.5 Other polymers (i)

There are no such residues in this entry.

CONFIDENTIAL VALIDATION REPORT
